# Intra-Amniotic Administration—An Emerging Method to Investigate Necrotizing Enterocolitis, In Vivo (*Gallus gallus*)

**DOI:** 10.3390/nu14224795

**Published:** 2022-11-12

**Authors:** Nikolai Kolba, Jacquelyn Cheng, Cydney D. Jackson, Elad Tako

**Affiliations:** Department of Food Science, Cornell University, Stocking Hall, Ithaca, NY 14850, USA

**Keywords:** necrotizing enterocolitis, NEC, dextran sodium sulfate, intraamniotic administration, *Gallus gallus*, gut microbiome, dysbiosis, intestinal immaturity

## Abstract

Necrotizing enterocolitis (NEC) is a severe gastrointestinal disease in premature infants and a leading cause of death in neonates (1–7% in the US). NEC is caused by opportunistic bacteria, which cause gut dysbiosis and inflammation and ultimately result in intestinal necrosis. Previous studies have utilized the rodent and pig models to mimic NEC, whereas the current study uses the in vivo (*Gallus gallus*) intra-amniotic administration approach to investigate NEC. On incubation day 17, broiler chicken (*Gallus gallus*) viable embryos were injected intra-amniotically with 1 mL dextran sodium sulfate (DSS) in H_2_O. Four treatment groups (0.1%, 0.25%, 0.5%, and 0.75% DSS) and two controls (H_2_O/non-injected controls) were administered. We observed a significant increase in intestinal permeability and negative intestinal morphological changes, specifically, decreased villus surface area and goblet cell diameter in the 0.50% and 0.75% DSS groups. Furthermore, there was a significant increase in pathogenic bacterial (*E. coli* spp. and *Klebsiella* spp.) abundances in the 0.75% DSS group compared to the control groups, demonstrating cecal microbiota dysbiosis. These results demonstrate significant physiopathology of NEC and negative bacterial–host interactions within a premature gastrointestinal system. Our present study demonstrates a novel model of NEC through intra-amniotic administration to study the effects of NEC on intestinal functionality, morphology, and gut microbiota in vivo.

## 1. Introduction

In premature infants, a leading gastrointestinal disease, necrotizing enterocolitis (NEC), accounts for approximately 2–13% of preterm and very-low-birth-weight (VLBW, <1500 g) infants in the United States [1,2,3]. Variations in incidences are attributed to different risk factor profiles, such as differing populations, detection rates, and inclusion and exclusion criteria for the disease [4,5,6,7]. Currently, there is no global incidental rate on NEC. Previous literature suggests that NEC is caused by intra-luminal pathogenic bacteria disrupting the intestinal villi, which upregulates inflammatory pathways, causing dysbiosis, and ultimately results in intestinal necrosis [8,9,10]. NEC is a multifactorial disease wherein symptoms start slowly, but decompensation occurs quickly, leading to fulminate NEC with pneumatosis intestinalis and portal gases [11,12,13,14]. The bacterial endotoxins released from opportunistic bacteria bind to Toll-like receptor four within epithelial cells, which activate pathogen-associated molecular pattern (PAMP) and release a complement and coagulation cascade effect within the immune system to break down gut mucosa [15,16,17]. Intestinal barrier disruption leads to bacteria entering intestinal cells and causes possible ischemia–reperfusion injury to the tissue [18].

NEC was initially investigated and induced in rodent and pig models through hypoxic/hypothermic and/or surgical interventions to mimic the multifactorial nature of the human disease [6,19,20,21]. In 1974, Barlow et al. demonstrated the first NEC model in rats, where gut flora and lack of immunoglobulin A (IgA) from breast milk were found to be essential factors contributing to NEC-like injury [22]. Currently, model organisms for NEC include rodents, pigs, and gnotobiotic quail, each with distinct strengths and weaknesses in modeling NEC [23,24]. Rats modeling NEC have practical benefits such as a low cost and high resilience to stress compared with mice; however, rats lack biomolecular reagents such as antibodies, meaning specific genetic techniques cannot be utilized to understand the mechanisms of pathophysiology, unlike mice [24]. The mouse model has been used to demonstrate NEC prevention mechanisms but is limited, as reproducible data is not necessarily obtained [19,25,26,27]. Another model, pigs, which have a closer resemblance in size, physiology, and anatomy when compared to a premature infant [28,29,30], is costly to maintain, and genetic techniques are limited [20,31,32,33]. Additionally, piglet models utilize intestinal injury to induce NEC, affecting the whole GI tract, while human NEC occurs primarily in the distal small intestine [32]. Lastly, there is the quail model, which is practical for NEC because of their modest size; rapid, productive maturation; resilience to research manipulation; transgenic lines; fully sequenced genome; and availability for molecular manipulation [34,35,36,37].

The current study suggests an alternative and novel model for NEC, the chicken (*Gallus gallus*). The chicken has been a well-studied model organism since the last century due to genetic analysis in developmental biology, virology, oncology, and immunology [38]. Chickens has been utilized for several human diseases, including muscular dystrophy [39,40,41], bacterial infections [42,43,44,45], autoimmunity [46,47,48], cancer [49,50,51], the microbiome [52,53,54,55,56,57], and micronutrient deficiencies [58,59,60,61,62,63,64]. The external embryology of the chicken has been a leading system investigating vertebrate development using functional genomics and biochemistry to study diseases similar to NEC. We hypothesize that the intra-amniotic administration [64,65,66] of dextran sulfate sodium (DSS, a compound previously demonstrated to induce NEC) will lead to NEC development, causing clinical symptoms within the brush border membrane functionality, tissue morphology, and dysbiosis of the intestinal microbial populations.

## 2. Materials and Methods

### 2.1. Sample Preparation

Dextran sulfate sodium (>98%) (Catalog #J62101.14, molecular weight 165.19 g/mol, ThermoFisher, Waltham, MA, USA) was used for the intra-amniotic administration experiment. In addition, 4 kDa fluorescein isothiocyanate-dextran (FITC-Dextran, Catalog #SIAL-46944-100M, Sigma-Aldrich, St. Louis, MO, USA) was used for the intestinal permeability assay.

### 2.2. Animals and Study Design

Cornish cross-fertile broiler eggs (*n* = 59) were purchased from a hatchery (Moyer’s Chicks, Quakertown, PA, USA). The eggs were incubated under standard conditions at the Cornell University Animal Science poultry farm. All animal experiments were approved and performed in compliance with Cornell University IACUC (protocol code: 2020-0077).

#### Intra-Amniotic Administration

Pure DSS solutions were individually diluted in deionized (DI) water. As previously described [54,56,61,62,63,67], intra-amniotic administration was completed on day 17 of embryonic development with viable embryos (*n* = 60). Eggs were weighed and divided into six treatment groups of equal weight distribution (*n* = 10), using a random sequence generation [68]. For the intra-amniotic administration, all eggs were disinfected by spraying 70% ethanol. In the H_2_O control and DSS-treated groups, a 21-gauge needle was inserted into the amniotic fluid, and 1 mL of sterile solution was injected. The site for intra-amniotic administration was identified via candling. After the administration, the injection sites were sprayed with 70% ethanol and sealed with transparent tape. The eggs were distributed into six groups: (1) no injection, (2) DI H_2_O, (3) 0.10% DSS, (4) 0.25% DSS, (5) 0.50% DSS, and (6) 0.75% DSS. The eggs were equally distributed in each incubator to reduce possible allocation bias. Upon hatch, on incubation day 21, chicks were euthanized by CO_2_. The blood was obtained via cardiac puncture and stored at 4 °C, then fractionated and stored at −20 °C. The proventriculus, gizzard, liver, pectoral muscle, duodenum, and cecum were obtained, flash frozen in liquid nitrogen, and stored at −20 °C until analysis.

### 2.3. Intestinal Permeability Test: Fluorescein Isothiocyanate Dextran (FITC-Dextran) Test

The intestinal permeability of the hatchlings was determined on day of hatch, as previously described by Barekatain et al., 2019 [69]. Briefly, on the day of hatch, each bird was orally gavaged with a 0.5 mL aqueous solution containing 1.1 mg of fluorescein isothiocyanate dextran (FITC-Dextran) before euthanization. A blood sample was taken from each bird after 4 h via myocardial puncture. Blood samples were fractionated via centrifugation at 1000× *g* for 15 min (Allegra X-30R, Beckman Coulter, Brea, CA, USA) and kept at −20 °C until analysis. Plasma samples and standards were analyzed in triplicate for FITC-Dextran concentration using a Biotek Epoch Microplate Spectrophotometer (Agilent Technologies, Santa Clara, CA, USA) with excitation and emission wavelengths set at 485 and 530 nm, respectively.

### 2.4. Glycogen Analysis as a Measurement of Energetic Status

All procedures were conducted as previously described [54,56,70]. A total of 20 mg of the liver was collected for glycogen analysis. Hepatic glycogen content was determined by multiplying the weight of the tissue by the amount of glycogen per 1 g of wet tissue.

### 2.5. Isolation of the Total RNA from the Duodenum Samples

As previously described [54,56,67], a RNeasy Mini Kit (Catalog #74106, Qiagen Inc., Valencia, CA, USA) utilized 30 mg of duodenal tissue (*n* = 5) to extract the total RNA according to the manufacturer’s protocol. Total RNA was eluted in 50 µL of RNase-free water. All steps were carried out under RNase-free conditions. RNA was quantified by absorbance at 260/280 nm, and the integrity of the RNA was verified by 1.5% agarose gel electrophoresis followed by ethidium bromide staining. RNA was stored at −80 °C.

### 2.6. Real-Time Polymerase Chain Reaction (RT-PCR)

To create the cDNA, a 20 µL reverse transcriptase (RT) reaction was completed in a BioRad CFX1000 Touch thermocycler (BioRad, Hercules, CA, USA) using the Improm-II Reverse Transcriptase Kit (Catalog #A1250; Promega, Madison, WI, USA). The concentration of the cDNA obtained was determined by measuring the absorbance at 260/280 nm with an extinction coefficient of 33 (single-stranded DNA) by a NanoDrop 1000 Spectrophotometer (ThermoFisher Scientific, Waltham, MA USA). A RT-PCR assay assessed genomic DNA contamination for the genetic samples [67,71].

### 2.7. Intestinal Primer Design and Real-Time Quantitative PCR Design

The primers used in the RT-qPCR were designed based on ten gene sequences from the Genbank database, using Real-Time Primer Design Tool software (IDT DNA, Coralvilla, IA, USA). The sequences and the description of the primers used in this work are found in Table 1. The *Gallus gallus* 18s rRNA primer was designed as the reference gene, and the results obtained from the qPCR system were used to normalize the primers listed in Table 1. As previously described [62,63,72,73], all real-time quantitative PCR procedures were conducted with the specific primers listed in Table 1.

### 2.8. Intestinal Content DNA Isolation, Bacterial Primer Design, and PCR Amplification of Bacterial 16S rDNA

Frozen cecal contents were placed into a sterile tube containing 9 mL of phosphate-buffered saline (PBS) (Catalog#75800-998, VWR, Radnor, PA, USA) and homogenized with silicone bead-beating for 3 min [67,75,76]. All procedures were conducted as previously described [63,67,72].

As previously described [76,77,78,79], primers for *Lactobacillus*, *Bifidobacterium*, *Clostridium*, *Escherichia coli*, and *Klebsiella* were used with a universal primer variable region in bacterial 16S rRNA and were used as an internal standard. The PCR products were loaded on 2% agarose gel, stained with ethidium bromide, and quantified by Quantity-One 1D analysis software version 4.6.8 (BioRad, Hercules, CA, USA). The results were given by proportions of each bacterial group compared to the universal primer, giving relative abundance as previously conducted and demonstrated, with primers listed in Table 2 [62,63,72,73,80].

### 2.9. Morphological Examination

As previously described [54,65,66,67,81,82,83,84,85], intestinal samples (duodenum) were collected after the study and fixed in 4% (*v*/*v*) buffered formaldehyde. The samples were fixed further in 4% (*v*/*v*) buffered formaldehyde, dehydrated, cleared, and embedded in paraffin. The duodenum tissue was cut into 5 µm sections and placed on positively charged slides. Sections were: deparaffinized in xylene, rehydrated in different concentrations of alcohol, and stained. Periodic acid–Schiff and Alcian blue were used to distinguish neutral (magenta) and acidic (blue) mucins. Four sections of the duodenum per chick (*n* = 5 per treatment group) were examined. Villus height, villus width, crypt depth, goblet cell number, and goblet cell diameter were measured in each segment, using light microscopy with CellSens Standard version 3.2 (Olympus Corporation, Tokyo, Japan). Villi height was measured using the lamina propria as the base; villi width, the depth of the crypt, and the number of goblet cells were counted per side of a cross-sectional view through the villus; goblet cell size was measured as the diameter of the goblet cells (µm^2^). Villi surface area was calculated from the villus height and width at half height according to Uni et al. [86] and calculated using the following equation:

(1) $$Villus\;surface\;area=2\prod \;\times \frac{VW}{2}\times VL$$

where *VW* is the average of three measurements of villus width, and *VL* is the villus length [87]. For the Alcian Blue and periodic acid–Schiff stain, the segments were counted for the types of goblet cells in the villi epithelium and goblet cells within the crypts. Goblet cells were counted in ten randomly selected villi or crypts per intestinal section (four intestinal sections per subject, 40 villi or crypts counted per subject). Goblet cell type was identified based on color, as periodic acid–Schiff and Alcian blue stains distinguishes between neutral (magenta), mixed (purple), and acidic (blue) mucins. Paneth cells were identified by their triangular shape within 10 randomly selected crypts per intestinal section and then counted and measured. The means were utilized for statistical analysis.

### 2.10. Statistical Analysis

Experimental treatments for the in ovo assay were arranged entirely randomly. The Shapiro–Wilk test was used to assess for normality. Statistical analyses were performed using one-way Analysis of Variance (ANOVA). Data is presented as means and standard deviations. Differences were considered significant at *p* < 0.05 using a post hoc Duncan or Tukey test was used to compare different NEC severity treatments, as described in figure or table legends. Statistical analysis was conducted using SPSS version 27.0 software (IBM, Armonk, NY, USA).

## 3. Results

### 3.1. Gross Physical Findings

There was a total hatchability rate of 95%. As shown in Table 3, there was no significant difference between body weight observed between DSS treatment groups and the controls. However, the cecum weight in the DSS treatment groups (0.1% and 0.5%) was significantly higher compared to the no-injection group (*p* < 0.05, Table 2).

Additionally, intra-abdominal abscesses were found within the proventriculus and gizzard within only the 0.5% and 0.75% DSS treatments (depicted in Supplementary Figure S1).

### 3.2. Hb Concentration and Hepatic Glycogen Levels

The Hb value in the 0.75% DSS treatment group was significantly higher than the water injected, 0.1% DSS, and 0.25% DSS treatment groups (Table 4). Furthermore, there was a significant (*p* < 0.05) difference between the 0.75% DSS group and all other hepatic glycogen treatment groups.

### 3.3. Change of Intestinal Permeability across the Groups

**Figure 1:**
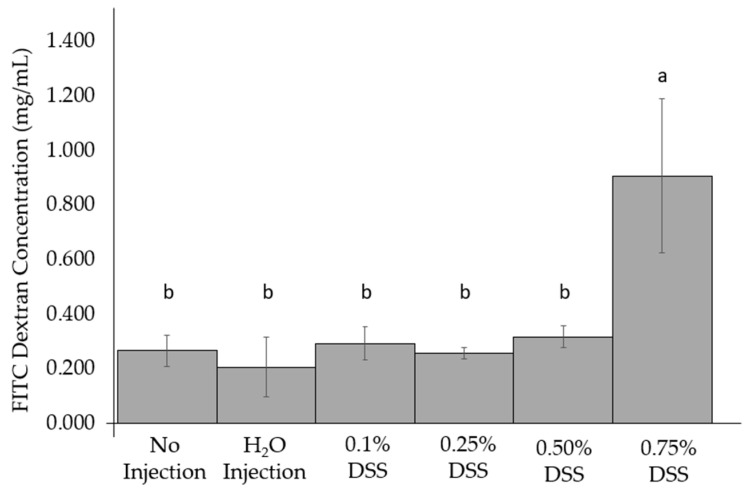
Comparison of the intra-amniotic administration of DSS to controls on the day of hatch within the small intestine (duodenum). Values are means ± stand error, *n* = 3. ^a,b^ within a column, means without a common letter are significantly different, *p* < 0.05 (Duncan’s post-hoc test).

DSS treatments were not significantly different from the non-treated FITC-dextran birds. However, the 0.75% DSS treatment group was significantly (*p* < 0.05, Figure 1) different than the other treatment groups treated with FITC-dextran. Furthermore, no dose-response occurred from the titration of concentrations between the experimental groups and controls.

### 3.4. Duodenal Gene Expression

**Figure 2:**
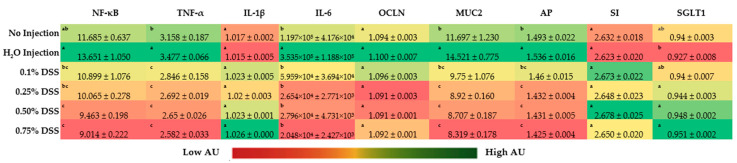
Effect of the intra-amniotic administration of DSS on intestinal gene expression on the day of hatch within the small intestine (duodenum). Values are means ± stand error, *n* = 5. ^a,b,c^ within a column, means without a common letter are significantly different, *p* < 0.05 (Duncan’s post-hoc test). NF-κβ, nuclear factor kappa-light-chain-enhancer of activated B cells; TNF-α, tumor necrosis factor-alpha; IL-1β: interleukin one beta; IL-6: interleukin 6; OCLN: occludin; MUC2, mucin 2; AP: aminopeptidase; SI: sucrose isomaltase; SGLT1: sodium-glucose transporter 1; 18s rRNA: 18S ribosomal subunit.

The gene expression of the inflammatory marker, NF-κβ1, was lower (*p* < 0.05) in the 0.50% and 0.75% DSS treatment groups compared to the control groups (no injection and H_2_O injection) (Figure 2). However, other concentrations of DSS did not affect the expression of NF-κβ1 (*p* < 0.05). The relative expression of TNF-α was significantly (*p* < 0.05) decreased in all of the DSS experimental groups (0.1%, 0.25%, 0.5%, and 0.75%) compared to the controls. Similarly, IL-6 was lowered in all of the DSS-treated groups compared only to the H_2_O injection group (Figure 2). However, no significant differences in IL-1β expression were found between any of the groups.

The gene expression of brush border membrane functionality proteins, sucrose isomaltase (SI), and occludin (OCLN) were not significantly different. Despite no significant difference in OCLN gene expression, there was a trend of decreased gene expression with increased DSS treatment concentration (Figure 2). There was a significant (*p* < 0.05) downregulation of MUC2 and AP gene expression in the DSS treatment groups compared to the H_2_O and no-injection control. There is a significant increase (*p* < 0.05) in the gene expression of SGLT1 in the 0.25%, 0.50%, and 0.75% DSS-treatment groups compared to the H_2_O-injected group.

### 3.5. Microbial Dysbiosis

**Figure 3:**
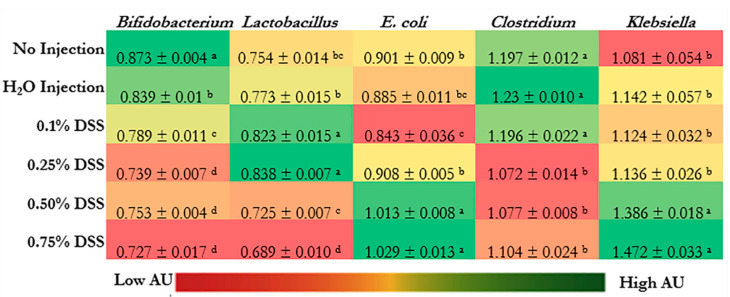
Effect of the intra-amniotic administration of DSS on cecal bacterial populations (day of hatch). Values are means ± SEM. Per bacterial category, ^a–d^ within a column, treatment groups that do not share letters are significantly different according to one-way ANOVA with Tukey’s post-hoc test (*p* < 0.05).

Figure 3 shows cecal bacterial populations. The relative abundance of *Bifidobacterium* spp. was significantly decreased in the DSS-treated groups (*p* < 0.05) relative to the control groups (non-injected and H_2_O-injected groups). *Bifidobacterium* spp. and *Lactobacillus* spp. were significantly decreased in the 0.50% and 0.75% DSS groups compared to the non-injected and H_2_O-injected groups. The highest relative abundances of *Lactobacillus* spp. were in 0.1% and 0.25% DSS following the exposure compared to the controls.

*E. coli* and *Klebsiella* spp., opportunistic and possibly pathogenic bacteria, were significantly increased (*p* < 0.05) in the two highest concentrations of DSS (0.50% and 0.75%) compared with the non-injected and the H_2_O-injected groups. However, the relative abundance of *Clostridium* spp. was significantly (*p* < 0.05) lowered in 0.25%, 0.50%, and 0.75% DSS compared to the control groups (non-injected and H_2_O-injected groups) and 0.1% DSS group.

### 3.6. Intestinal Morphology

The villus surface area and crypt goblet cell diameter were significantly (*p* < 0.05) lowered in the 0.75% DSS group compared to the H_2_O injection group (Table 5, images withing Supplemental Figure S2), indicating that DSS negatively impacted intestinal development. A significant (*p* < 0.05, Table 5) increase was found in the villi goblet cell diameters, Paneth cell number, and Paneth cell diameter of 0.1% and 0.75% DSS groups compared to the non-injected and H_2_O-injected groups. There was no significant difference in crypt depth between experimental groups.

A closer investigation of goblet cells within crypts and villi was viewed to determine differences (Table 6). Crypt goblet cell count was significantly increased in the 0.1% and 0.75% DSS groups compared to the non-injected and H_2_O injection groups. Different goblet cell types were analyzed in crypts; acidic goblet cells were significantly higher (*p* < 0.05) in the 0.1% and 0.75% DSS groups compared to the control groups. There was a significantly (*p* < 0.05) higher amount of mixed goblet cells in crypts within the 0.1% DSS group compared to the control groups. Similarly, the villi goblet cell number and the acidic and mixed goblet cell number were significantly increased with 0.1% DSS exposure compared with the controls, as seen in Table 6.

## 4. Discussion

NEC is an acute inflammatory disease that results in the intestinal necrosis of the bowels, systemic sepsis, and multiorgan failure from a complex combination of pathological events, including patchy inflammation of the small intestine and intestinal hypoxic/reperfusion injuries and bacterial dysbiosis [3,13,16,88]. In our present study, we investigated the effects of dextran sodium sulfate (DSS) utilizing the *Gallus gallus* intra-amniotic administration model to mimic necrotizing enterocolitis (NEC). The present study indicates that the intra-amniotic administration of DSS at the highest concentration (0.75%) had similar findings as NEC manifestations in humans, including but not limited to the inflammation of the small intestines, intra-abdominal abscesses of the gizzard, increased hemoglobin levels, increased permeability within the intestines, and the increased presence of potentially pathogenic bacteria. NEC has previously been induced and investigated in the rodent and pig models through hypoxic/hypothermic and/or surgical interventions to simulate the multifactorial nature of NEC in humans [6,19,20,21].

Current NEC in vivo models, such as rodents, pigs, and quails, have distinct strengths and weaknesses. Rodents (mice and rats) are induced to have NEC by cesarian section delivery before term, then gavage fed with the formula [19,89,90]. Mice have antibodies, and specific genetic techniques can be utilized to understand NEC pathophysiology mechanisms, whereas rats lack antibodies but are more resilient to stress [24]. Unfortunately, studies using the mouse model have shown inconsistent results, wherein data reproducibility presents potential issues [19,25,26,27]. While pigs have closer resemblance relative to rodents in physiology, there are drawbacks in which NEC is induced via intestinal injury to the whole intestine, and genetic techniques also present limitations. In another model, gnotobiotic quails, NEC is induced via the oral gavage of bacteria associated with NEC to affect its small intestine, which has aided in understanding the inducible nitric oxide synthase (iNOS) pathway before macroscopic lesions [34,35,91]. Quails have been shown to be an NEC model that balances practicality, resilience, and molecular manipulation [34,35,36,37]. Given these aforementioned factors and that the chicken model has been a leading system investigating vertebrate development using functional genomics and biochemistry to study diseases similar to NEC [38], we sought to develop another potential model for NEC using the embryonic stage of the *Gallus gallus*.

In our experimental trials, the intra-amniotic administration of DSS was utilized to induce intestinal inflammation to cause NEC physiopathology [92,93,94]. DSS is a sulfated polysaccharide with various molecular weights (5–1400 kDa), commonly used to induce enteric colitis in rodents by penetrating the intestinal mucosal membrane [19,93,94,95,96]. DSS-induced colitis is a widely used model because it is rapid, simple, reproducible, and controllable. Recent studies have shown that DSS added to DI H_2_O induces clinical, gross, and histological factors associated with enteritis in broiler chickens, such as decreased body weight, bloody diarrhea, intestinal lesions, shortened villi height, and increased goblet cell density [92,94,97,98]. Furthermore, Zou et al. (2018) demonstrated that DSS exposure to broiler chickens increases gut leakiness and induces pro- and anti-inflammatory cytokine response elements in a dose-dependent manner [92]. Nevertheless, there is limited research characterizing DSS-induced NEC in *Gallus gallus*, and this study demonstrates the first-ever intra-amniotic administration of DSS to induce NEC.

During the initial necropsies, there was little to no inflammation within the internal organs of the chicks on the day of hatch in the 0.1, 0.25, and 0.5% DSS exposed groups. However, the 0.75% DSS treatment group had a few slightly patchy inflammation sites in the small intestines and showed intra-abdominal abscesses within the proventriculus and gizzard (Supplemental Figure S1); these observations are in agreement with previous studies that demonstrated the initial hypoxic and reperfusion injuries (typical in NEC cases from rodents and clinical models) and an increase in innate immune responses (cytokines and white bloods cells). [11,20,24,92,99]. The intra-abdominal abscesses and patchy inflammation in the distal digestive organs resulting from the treatment with the highest DSS concentration (0.75%) can be associated with hypoxic reperfusion injuries, which have previously been demonstrated with DSS exposure [92,94,95]. To further illustrate the effectiveness of DSS penetrating through the mucosal layers of the small intestine, a FITC-dextran assay was performed (Figure 1). Compared to all other groupings, there was a significant (*p* < 0.05) increase in the intestinal mucosal layer penetration in the 0.75% DSS treatment group. The increased intestinal permeability suggests that the DSS successfully breaks down the mucosal layer, potentially allowing pathogenic bacteria to invade the host’s villi, as previously described [9,25,69,100,101]. However, occludin (OCLN) gene expression, a tight junction protein between the intestinal enterocytes, does not significantly differ between the groups (Figure 2). The lack of significance of the gene expression of the tight junctions on the basolateral surface could be the short duration of a DSS exposure time to allow for occludin degradation. Though no significant alterations in OCLN gene expression were found, our results supporting increased intestinal permeability with DSS exposure were further supported by our hemoglobin and hepatic glycogen results. The hemoglobin concentration was raised within the DSS groups compared to the non-injected group (Table 4). As intestinal irritability and instability increase, hemoglobin values were found to increase in clinical patients with inflammatory bowel disease [102]. Hepatic glycogen levels were increased only within the 0.75% DSS group compared to others (Table 4). However, Sodhi et al. (2009) demonstrated that enterocyte proliferation is inhibited in rat intestinal cell lines (IEC-6 cells enterocytes) and TLR4^-/-^ mice and that glycogen synthase kinase decreases when under NEC conditions with lipopolysaccharides [103]. This observed difference could be due to the treatment to induce the condition and the difference between the models utilized.

Brush border membrane (BBM) functionality was investigated by measuring the gene expression of the functional proteins viewing the digestive capabilities, as seen in Figure 2. There was no significant difference between the treatment groups in sucrose isomaltase or sodium–glucose transporter 1. However, there was a substantial lowering of mucin 2 (MUC2) and aminopeptidase (AP) gene expression in the DSS treatment groups (0.25%, 0.50%, and 0.75%) compared to the no-injection and H_2_O-injection groups. As previously mentioned, DSS is a sulfated polysaccharide that disrupts the luminal mucus layer, allowing mucosal thinning and opportunistic bacteria to penetrate the BBM, causing intestinal trauma [104,105]. This reasoning on MUC2 supports the findings on the lowering of aminopeptidase expression, as AP is primarily located near the apical side of the lumen in the intestinal epithelial cells [106]. Aminopeptidases are enzymes that catalyze the amino terminus of a protein within subcellular organelles, cytosol, and membrane components. Thus, it can be suggested that, if the BBM membrane is injured, the AP capacity would be significantly reduced, increasing the possibility of immune responses (pro-inflammation and apoptosis) [107].

Inflammation resulting from NEC is a primary identifier of injury from hypoxia/reperfusion conditions and an indicative marker for the disease [15,32,108,109]. Inflammation biomarkers, nuclear factor kappa-light-chain-enhancer of activated B cells (NF-κβ), tumor necrosis factor-alpha (TNF-α), interleukin one beta (IL-1β), and interleukin 6 (IL-6) were all analyzed via RT-qPCR (Figure 2) due to the use of these biomarkers in other NEC models and clinical trials [108,109,110,111,112]. NF-κβ and TNF-α were significantly (*p* > 0.05) lower in the 0.50% and 0.75% DSS groups compared to the control groups (non-injected and H_2_O-injected). The downregulation of NF-κβ gene expression originates from the upstream signaling of TNF-α being downregulated since these proteins operate in tandem with pro-inflammation pathways triggered by microbial products (i.e., endotoxins, metabolites, amino acids, etc.) and signal transductions mechanisms in the innate immune system [113,114,115,116,117]. The downregulation of TNF-α is potentially derived from the upregulation of microbial byproducts within the duodenum in the DSS treatments. Krishnaveni and Jayachandran (2009) found that ethyl acetate extracts from two different marine bacteria caused the downregulation of TNF-α [118]. Similarly, Lou et al. (2018) found that *Brucella* caused the same downregulation of TNF-α within porcine and murine models [119].

There were significant changes within the bacterial profiles in the DSS treatment groups (Figure 3). Bacterial profiles of the 0.75% DSS group were significantly (*p* < 0.05) lower abundance of *Bifidobacterium*, *Lactobacillus,* and *Clostridium* spp. In contrast, the DSS-treatment groups demonstrated significantly higher abundance levels of *E. coli* and *Klebsiella* spp. (*p* < 0.05). The lower abundance of beneficial bacteria (Bifidobacterium and Lactobacillus) suggests an opportunity for dysbiosis via the proliferation of opportunistic bacteria such as *E. coli* and *Klebsiella*. Our results are similar to other NEC models that utilized different treatments to induce NEC [6,9,17,20,24,26,34,91,120]. One of the first NEC models used *Klebsiella* to create an NEC model (mice) because the genus produces hydrogen-sulfide-rich gas pockets of pneumatosis in vivo [22,121]. Similarly, it is theorized that gas produced by *E. coli* can invade the same intraluminal cavities as *Klebsiella,* which leads to pneumatosis intestinalis, which is a radiographic sign of NEC [122,123,124,125]. Additionally, Tarracchini et al. (2021) found that *E. coli* and other opportunistic bacteria are found within the next-generation sequencing of NEC clinical patients, suggesting that the bacterial abundance of *E. coli* could induce NEC pathology [126]. These invasive bacterial changes the grouping/profile of the gut and influences the intestinal BBM morphology [126,127,128,129,130,131,132].

Within this study, the duodenum was sectioned to investigate DSS’s effect on its intestinal morphology (Supplemental Figure S2). Previous studies have shown that NEC results in various levels of intestinal degradation due to microbial dysbiosis effects on the brush border membrane morphology (i.e., the villi surface area, goblet cell number, type, size, Paneth cell production) [133,134,135]. The 0.75% DSS treatment group had significantly lower villus surface area, crypt goblet cell diameter, and villi goblet cell number and type. In parallel, there was a significant increase in the Paneth cell number and size (Table 5) and crypt goblet cell number and types (Table 6) populations. Since goblet cells produce mucin, which lubricates the passage of food through the intestines and protects the intestine from the potential damage from digestive enzymes, the DSS treatments at the highest concentration would be associated with damage at the apical side of the enterocyte while lowering the villus surface area, which aligns with previous DSS studies and is in agreement with the present study [136,137,138]. Additionally, on the enterocyte’s basolateral side, the crypts’ goblet cells would anticipate the loss of mucin and increase its mucin production to overcome the loss [139,140]. This anticipation can be further supported by the increase in Paneth cell findings within the crypts of the intestinal epithelial cells (*p* < 0.05, Table 5). Paneth cells within the small intestine synthesize and secrete antimicrobial enzymes as a part of the innate immune system [100,141,142]. The antimicrobial peptide secretion by Paneth cells is recognized by MyD88-dependent Toll-like receptor (TLR) activations, triggering the expression of multiple peptides and proteins [19,25]. The Paneth cells migrate towards the base of the villi after differentiation within the crypts to protect commensal bacteria from the opportunistic bacteria within the gut, which supports the findings of bacterial dysbiosis mentioned earlier in Figure 3.

## 5. Conclusions

This study is the first to demonstrate NEC symptoms via the intra-amniotic administration of DSS in vivo (*Gallus gallus*). The 0.75% DSS treatment group decreased BBM functionality and demonstrated microbiota dysbiosis within a premature gut, mimicking other models of NEC. Although we did not observe significant severe pathologies (gas-filled lesions or necrotic plaques in histological sectioning), there was a clear trend of opportunistic bacterial populations proliferation and overtaking the distal gastrointestinal tract. This transformation of untreated and DSS-treated individuals’ microbial profiles can potentially affect several bacterial metabolic pathways related to bacterial, cellular, and metabolic processes. The results of this study are promising evidence to investigate increased concentrations of DSS to cause more severe NEC symptoms and identify potential novel biomarkers for less severe NEC cases. Furthermore, the suggested in vivo novel model and innovative approach will support the assessment of various potential interventions to ameliorate the pathophysiology of NEC.

## Figures and Tables

**Table 1:** Primer sequences used in the study.

Target Gene	Forward (5′-3′)	Reverse (3′-5′)	Amplicon Length (Base Pairs)	NCBI Accession	Ref.
Inflammatory Genes					
NF-κβ	CACAGCTGGAGGGAAGTAAAT	TTGAGTAAGGAAGTGAGGTTGAG	100	2130627	
TNF-α	GACAGCCTATGCCAACAAGTA	TTACAGGAAGGGCAACTCATC	109	53854909	
IL-1β	TCATCCATCCCAAGTTCATTCA	GACACACTTCTCTGCCATCTT	105	395872	
IL-6	ACCTCATCCTCCGAGACTTTA	GCACTGAAACTCCTGGTCTT	105	302315692	
Brush Border Membrane (BBM) Functionality Genes					
OCLN	GTCTGTGGGTTCCTCATCGT	GTTCTTCACCCACTCCTCCA	124	396026	[74]
MUC2	CCTGCTGCAAGGAAGTAGAA	GGAAGATCAGAGTGGTGCATAG	272	423101	
AP	CGTCAGCCAGTTTGACTATGTA	CTCTCAAAGAAGCTGAGGATGG	138	45382360	
SI	CCAGCAATGCCAGCATATTG	CGGTTTCTCCTTACCACTTCTT	95	2246388	
SGLT1	GCATCCTTACTCTGTGGTACTG	TATCCGCACATCACACATCC	106	8346783	
18S rRNA	GCAAGACGAACTAAAGCGAAAG	TCGGAACTACGACGGTATCT	100	7262899	

NF-κβ, nuclear factor kappa-light-chain-enhancer of activated B cells; TNF-α, tumor necrosis factor-alpha; IL-1β: interleukin one beta; IL-6: interleukin 6; OCLN: occludin; MUC2, mucin 2; AP: aminopeptidase; SI: sucrose isomaltase; SGLT1: sodium-glucose transporter 1; 18S rRNA: 18S ribosomal subunit. Target genes were created from accessions within National Center for Biotechnology Information (NCBI).

**Table 2:** Microbial primer sequences for bacteria within cecum.

Target Gene	Forward (5′-3′)	Reverse (3′-5′)	Ref.
*Lactobacillus* spp.	CATCCAGTGCAAACCTAAGAG	GATCCGCTTGCCTTCGCA	[77]
*Bifidobacterium* spp.	GGGTGGTAATGCCGGATG	CCACCGTTACACCGGGAA	[77]
*E. coli* spp.	GACCTCGGTTTAGTTCACAGA	CACACGCTGACGCTGACCA	[77]
*Clostridium* spp.	AAAGGAAGATTAATACCGCATAA	ATCTTGCGACCGTACTCCCC	[77]
*Klebsiella* spp.	CGCGTACTATACGCCATGAACGTA	ACCGTTGATCACTTCGGTCAGG	[78,79]
16S rRNA	CGTGCCAGCCGCGGTAATACG	GGGTTGCGCTCGTTGCGGGACTTAACCCAACAT	[77]

**Table 3:** The effect of DSS on the body weight, cecum weight, and cecum-to-body-weight ratio.

Group	Body Weight (g)	Cecum Weight (g)	Cecum: Body Weight
No Injection	40.06 ± 4.06 ^b^	0.42 ± 0.06 ^b^	0.015 ± 0.005 ^a^
H_2_O Injection	47.49 ± 1.21 ^a^	0.47 ± 0.03 ^a,b^	0.010 ± 0.001 ^a^
0.1% DSS	45.81 ± 1.23 ^a,b^	0.62 ± 0.08 ^a^	0.013 ± 0.002 ^a^
0.25% DSS	45.25 ± 1.01 ^a,b^	0.49 ± 0.05 ^a,b^	0.011 ± 0.001 ^a^
0.50% DSS	45.24 ± 1.11 ^a,b^	0.64 ± 0.08 ^a^	0.014 ± 0.002 ^a^
0.75% DSS	45.78 ± 0.86 ^a,b^	0.60 ± 0.06 ^a,b^	0.010 ± 0.000 ^a^

Values are means ± stand error, *n* = 8–10. ^a,b^ within a column, means without a common letter are significantly different, *p* < 0.05 (Duncan’s post-hoc test).

**Table 4:** Blood hemoglobin (Hb) concentrations (g/dL) and hepatic glycogen levels (mg/mL).

Group	Hb (g/dL)	Hepatic Glycogen (mg/mL)
No Injection	10.48 ± 1.31 ^a^	0.002 ± 0.001 ^b^
H_2_O Injection	9.82 ± 0.77 ^a^	0.003 ± 0.001 ^b^
0.1% DSS	10.70 ± 1.16 ^a^	0.003 ± 0.001 ^b^
0.25% DSS	10.22 ± 1.56 ^a^	0.004 ± 0.001 ^b^
0.50% DSS	10.13 ± 0.77 ^a^	0.004 ± 0.001 ^b^
0.75% DSS	10.94 ± 3.24 ^a^	0.008 ± 0.002 ^a^

Values are means ± standard error, *n* = 5. ^a,b^ within a column, means without a common letter are significantly different, *p* < 0.05 (Tukey’s post-hoc test).

**Table 5:** Effects on intestinal villi and crypts of the duodenum after the intra-amniotic administration of experimental DSS.

Treatment	Villus Surface Area (µm^2^)	Crypt Depth (µm)	Villi Goblet Diameter (µm)	Crypt Goblet Diameter (µm)	Paneth Cell ^#^	Paneth Cell Diameter (µM)
No Injection	109.99 ± 3.06 ^d^	25.17 ± 0.93 ^a,b^	3.57 ± 0.05 ^d^	2.99 ± 0.05 ^b^	1.09 ± 0.02 ^c^	1.56 ± 0.03 ^b^
H_2_O Injection	205.15 ± 5.03 ^a^	26.35 ± 0.98 ^a,b^	4.04 ± 0.06 ^c^	3.16 ± 0.04 ^a^	1.03 ± 0.01 ^c^	1.47 ± 0.02 ^c^
0.1% DSS	147.51 ± 3.28 ^b^	27.89 ± 1.08 ^a^	4.55 ± 0.07 ^a^	2.99 ± 0.04 ^b^	1.80 ± 0.05 ^b^	1.69 ± 0.03 ^a^
0.75% DSS	130.35 ± 0.03 ^c^	24.32 ± 0.78 ^b^	4.25 ± 0.05 ^b^	2.73 ± 0.04 ^c^	1.93 ± 0.06 ^a^	1.67 ± 0.03 ^a^

Values are means ± stand error, *n* = 5. ^a–d^ within a column means without a common letter are significantly different, *p* < 0.05 (Duncan’s post-hoc test). ^#^ Number of cells.

**Table 6:** Effects on intestinal villi and crypt goblet cells of the duodenum after the intra-amniotic administration of experimental DSS.

Treatment	Crypt Goblet Cell ^#^	Crypt Goblet Cell Type Number			Villi Goblet Cell ^#^	Villi Goblet Cell Type Number		
		Acidic	Neutral	Mixed		Acidic	Neutral	Mixed
No Injection	8.57 ± 0.32 ^c^	6.59 ± 0.26 ^c^	0.00 ± 0.0	1.97 ± 0.18 ^d^	15.78 ± 0.45 ^c^	13.7 ± 0.42 ^c^	0.00 ± 0.00	2.08 ± 0.13 ^c^
H_2_O Injection	7.96 ± 0.24 ^c^	7.42 ± 0.22 ^b^	0.00 ± 0.00	0.49 ± 0.06 ^c^	22.93 ± 0.6 ^b^	18.4 ± 0.53 ^b^	0.00 ± 0.00	4.53 ± 0.23 ^b^
0.1% DSS	14.7 ± 0.41 ^a^	10.23 ± 0.31 ^a^	0.00 ± 0.00	4.47 ± 0.19 ^a^	30.37 ± 0.84 ^a^	23.91 ± 0.68 ^a^	0.00 ± 0.00	6.46 ± 0.36 ^a^
0.75% DSS	13.48 ± 0.04 ^b^	9.76 ± 0.32 ^a^	0.00 ± 0.00	3.74 ± 0.19 ^b^	16.64 ± 0.56 ^c^	14.63 ± 0.49 ^c^	0.00 ± 0.00	2.09 ± 0.14 ^c^

Values are means ± stand error, *n* = 5. ^a–d^ within a column means without a common letter are significantly different, *p* < 0.05 (Duncan’s post-hoc test). ^#^ Number of cells.

## Supplementary Materials

The following are available online at https://www.mdpi.com/article/10.3390/nu14224795/s1, Figure S1: Representative images of gross anatomical photos of dissections and proventriculus/gizzards of birds on the day of hatch; Figure S2. Representative histology (Alcian Blue and Pacific Acid Schiff staining) images of the duodenum of birds on the day of hatch.
