# Supplementary material for: Intra-Amniotic Administration—An Emerging Method to Investigate Necrotizing Enterocolitis, In Vivo (Gallus gallus)
Source: Nutrients. 2022 Nov 12;14(22):4795. doi: 10.3390/nu14224795 (PMC9696943; doi:10.3390/nu14224795)
Supplement: Supplementary file 1 [file nutrients-14-04795-s001.zip › nutrients-1993910-supplementary.pdf]

Supporting Information

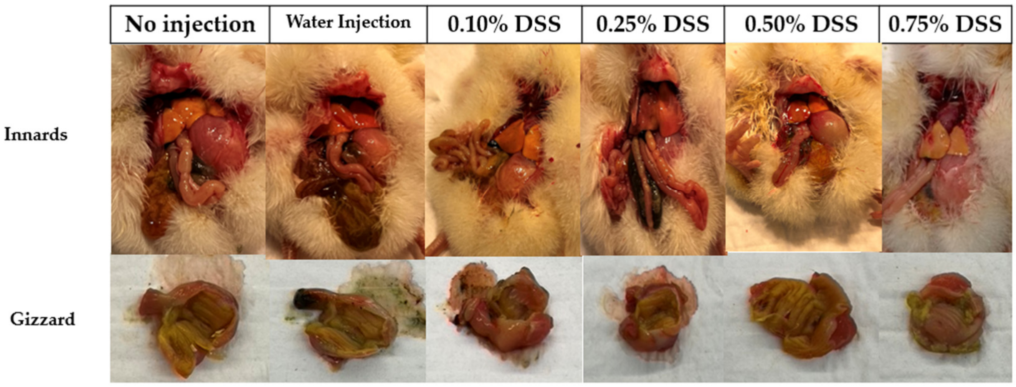

Figure S1. Representative images of gross anatomical photos of dissections and proventriculus/gizzards of birds on the day of hatch. The red circle demonstrates an intra-abdominal abscess within the gizzard of the 0.75% DSS treatment on the day of hatch. Yellow arrows illustrate the location of the duodenum, indicating decreasing anemia within the small intestine.

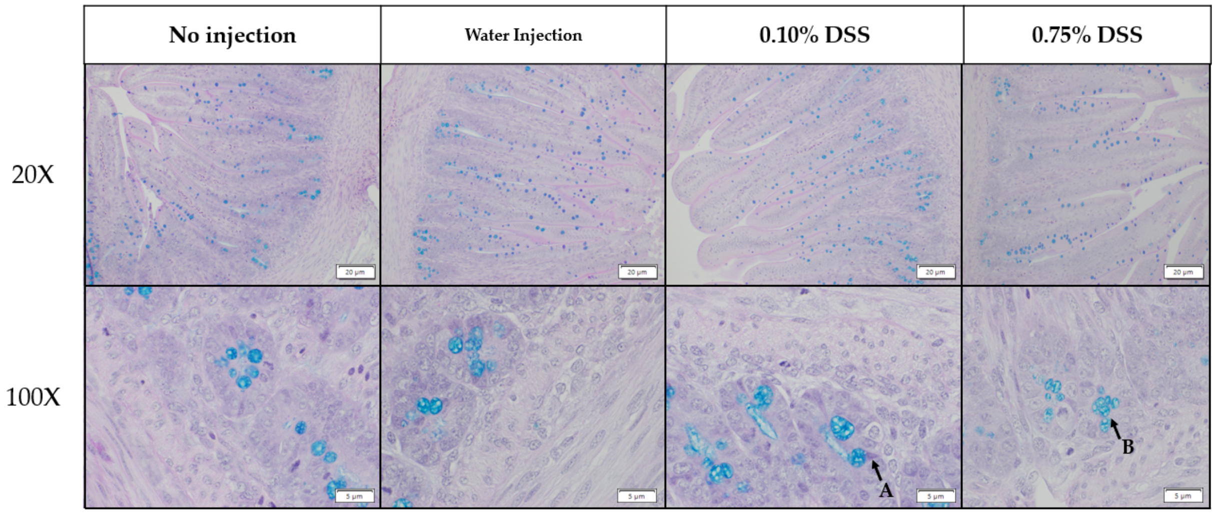

Figure S2. Representative histology (Alcian Blue and Pacific Acid Schiff staining) images of the duodenum of birds on the day of hatch. (A) Points out a Paneth cell that is stained light purple within a crypt. (B) Points to an acidic goblet cell, brilliant stained blue within a crypt.
